# Analysis of the components and pH of a sample of wet wipers used for the hygiene of newborns and infants^☆^^☆☆^

**DOI:** 10.1016/j.abd.2020.09.011

**Published:** 2021-09-15

**Authors:** Rosana Lazzarini, Mariana de Figueiredo Silva Hafner, Carolina Contin Proença, Luciana Rodino Lemes, Ana Carolina Rodrigues, Danielle Vieira Sobral

**Affiliations:** a Dermatology Clinic, Santa Casa de Misericórdia de São Paulo, São Paulo, SP, Brazil; b Faculty of Medical Sciences, Santa Casa de Misericórdia São Paulo, São Paulo, SP, Brazil; c Hospital Pedro Ernesto, Universidade do Estado do Rio de Janeiro, Rio de Janeiro, RJ, Brazil

Dear Editor,

Newborns and infants are susceptible to the occurrence of contact dermatitis in the diaper area, both in the irritative (ICD) and allergic (ACD) forms, due to constant exposure to their fecal waste, urine, and hygiene products.^1^

In this context, the use of wet wipers allows the cleaning of children regardless of the circumstances, speeding up and facilitating its practice in any location. These products consist of pieces of fabric soaked in an aqueous emulsion or oily lotion. Their use, however, can lead to adverse events and, therefore, care should be taken regarding their composition.^2^

The aim of the present work was to evaluate the characteristics of wet wipes commercialized in the city of São Paulo, Brazil, through the analysis of their pH values and composition.

Forty-two samples of wet wipers were acquired (from different locations and at variable prices) and cataloged. Each brand was analyzed separately in a reference laboratory.

Centrifuge tubes with 14 mL filters (Amicon™ Ultra-4 Centrifugal Filters) were used. The wipers of each brand were cut and positioned in a porous membrane filter with ultrafiltration capacity, in order to fill it completely. Each tube was identified and centrifuged (Eppendorf Centrifuge 5810 R™) for 30 minutes at 4,000 rotations per minute, which was repeated five times. At the end of the process, the amount of 2 mL of the aqueous solution was obtained from each wet wipe, which was then submitted to pH analysis using an appropriately calibrated benchtop pH meter (QUIMIS™ - Q400AS).^3^

The obtained pH measurements ranged from 3.53 to 7.43 (Table 1). Values ​​between 5.5 and 7.0 were found in 18 products (43%), a range considered to be ideal, close to the pH of the skin. However, more than half of the samples (54.7%) showed values ​​below this level, and one sample was >7.0. Products with a pH different from that of the skin can cause changes in the skin barrier functions: the lower ones act as irritants, and the higher ones inhibit the activity of proteases, making lipid synthesis difficult.^4^

**Table 1:** pH measurements of the evaluated wet wipers.

Commercial brand	pH after centrifuging
Baby Ever Care lavender-scented	6.90
Baby Ever Care fragrance-free	4.54
Baby Ever Care with aloe vera	4.81
Be better baby with aloe vera	5.05
Baby ever care with chamomile (yellow color)	5.89
Be better baby fragrance-free for sensitive skin	5.20
Bebê Natureza	5.07
Baby Wipes	5.65
Baby sec super premium *Galinha Pintadinha*	6.45
Baby sec ultrafresh *Galinha Pintadinha*	4.63
Bummis Capricho	5.77
Cotton Line Bichos	6.62
Dove Baby	4.54
Dove Baby Sensitive	4.63
Dry baby plus	5.70
Enxutita by Capricho	6.20
Feel clean	6.28
Giovanna Baby	4.84
Granado	5.33
Huggies pure care	5.58
Huggies supreme care	5.54
Huggies one & done	6.02
Huggies classic	5.50
Johnson’s (orange color)	4.76
Johnson’s bedtime	4.72
Johnson’s newborn	5.18
Johnson’s fresh touch (green color)	5.53
Johnson’s skin protection (pink color)	5.33
Johnson’s baby time to play (blue color)	5.26
Johnson’s newborn hypoallergenic	5.11
Meu bebê ultra	5.76
Mustela	4.62
Natural baby	5.39
Natura	6.14
Needs	7.43
Pampers fresh clean	3.56
Pampers sensitive	3.98
Personalidade baby	4.70
Petty baby	5.65
Piquitucho	5.28
Qualitá	5.03
Vic baby	5.93

The packaging of the products was also analyzed to verify the composition described on the labels by the manufacturers, specifying surfactants, preservatives, and fragrances (Table 2, Table 3, Table 4).

**Table 2:** Surfactants found on the labels of evaluated products.

Surfactants	Number	%
Cocamidopropyl betaine	24	57.1
Polysorbate 20	15	35.7
Lauryl glucoside	07	16.6
Coco glucoside	07	16.6
PEG-40 castor oil	05	11.9
Sodium laureth sulfate	04	9.5
Bis PEG/PPG-16/16	02	4.7
C12-13 pareth 3	01	2.3
C12-13 pareth 23	01	2.3
Glyceryl stearate	01	2.3

**Table 3:** Preservatives found on the labels of evaluated products.

Preservative	Number	%
Phenoxyethanol	29	69.0
Sodium benzoate	16	38.0
Methylparaben	12	29.5
Methylisothiazolinone (MI)	09	21.4
Bronopol	06	14.2
DMDM hydantoin	04	9.5
Isobutylparaben	04	9.5
Butylparaben	04	9.5
Propylparaben	04	9.5
Methylchloroisothiazolinone (MCI)	03	7.1
Potassium Sorbate	03	7.1
Diazolidinyl urea	02	4.7
Ethylparaben	02	4.7

**Table 4:** Fragrances found on the labels of evaluated products.

Fragrance	Number	%
Perfume	29	69.0
Coumarin	14	33.0
Citronellol	13	30.1
Linalool	12	28.6
Geraniol	11	26.2
Limonene	08	19.0
Hexyl cinnamal	06	14.3
Citral	03	7.1
Hydroxycitronellal	03	7.1
Cinnamic alcohol	03	7.1
Eugenol	01	2.4
d-limonene	01	2.4

Surfactants are compounds added to hygiene products that have detergent and foaming power. The most common was cocamidopropyl betaine, present in 57.1% of the products. This is a substance with widespread use due to its low cost, good cleaning capacity, moderate antimicrobial activity, non-toxicity, and compatibility with different pHs. However, studies have demonstrated its allergenic capacity, including in children.^2^, ^5^

Preservatives are added to ensure durability, avoiding contamination after the package is opened. In the analyzed products, phenoxyethanol was the most commonly used. It is a safe preservative, with a broad antimicrobial spectrum and low capacity to induce ACD, recommended for products to be used in children. On the other hand, the presence of preservatives with high allergenic capacity was observed, such as methylisothiazolinone and formaldehyde-releasing agents (Bronopol, DMDM ​​hydantoin and Diazolidinyl urea).^6^

The presence of parabens was observed in several of the analyzed products. Although they are controversial because of their possible relationship with breast cancer, they have a low allergenic capacity.^2^ Their relationship with cancer has never been clarified and their use is allowed in Europe and the USA, since their estrogenic activity seems to be very low. In Brazil, their use is authorized by the Brazilian Health Surveillance Agency (*Agência Nacional de Vigilância Sanitária –* [ANVISA]) according to Resolution n. 29/2012.^7^, ^8^

Fragrances are products that often cause ACD. In this evaluation, 104 fragrances were found, with an average of 2.47 per product. The designation “perfume” was found in 29 (69%) products, a term used for components that do not require discrimination on the label as they are within the limits established by ANVISA, which, however, does not eliminate the risk for ACD. Only nine products (21.4%) were considered “fragrance-free”, after the components were evaluated.^2^, ^9^

Wet wipers represent a great advance as they are very practical for hygiene but they have the potential to cause adverse events. Thus, their use should be assessed, especially in atopic children or children with skin lesions in the diaper area.^10^

## Financial support

None declared.

## Authors' contributions

Rosana Lazzarini: Design and planning of the study; drafting and editing of the manuscript; collection, analysis, and interpretation of data; effective participation in research orientation; intellectual participation in the propaedeutic and/or therapeutic conduct of the studied cases; critical review of the literature; critical review of the manuscript; approval of the final version of the manuscript.

Mariana de Figueiredo Silva Hafner: Design and planning of the study; drafting and editing of the manuscript; collection, analysis, and interpretation of data; effective participation in research orientation; intellectual participation in the propaedeutic and/or therapeutic conduct of studied cases; critical review of the literature; critical review of the manuscript; approval of the final version of the manuscript.

Carolina Contin Proença: Effective participation in research orientation; intellectual participation in the propaedeutic and/or therapeutic conduct of studied cases; critical review of the literature; critical review of the manuscript; approval of the final version of the manuscript.

Luciana Rodino Lemes: Effective participation in research orientation; intellectual participation in the propaedeutic and/or therapeutic conduct of studied cases; critical review of the literature; critical review of the manuscript; approval of the final version of the manuscript.

Ana Carolina Rodrigues: Drafting and editing of the manuscript; collection, analysis, and interpretation of data; approval of the final version of the manuscript.

Danielle Vieira Sobral: Collection, analysis, and interpretation of data; approval of the final version of the manuscript.

## Conflicts of interest

None declared.
